# Metabolic Reprogramming of B Cells in Cancer: Effects of Altered Energetics

**DOI:** 10.3390/biology15100744

**Published:** 2026-05-08

**Authors:** Uday Aditya Sarkar, Naqiya Ambareen, Parash Prasad, Mohd Kamran, Sampurna Ghosh

**Affiliations:** 1 Department of Medical Oncology, Dana-Farber Cancer Institute, Boston, MA 02215, USA; parash_prasad@dfci.harvard.edu (P.P.); fnu_mohdkamran@dfci.harvard.edu (M.K.); 2 Department of Medicine, Harvard Medical School, Boston, MA 02115, USA; nambareen@mgh.harvard.edu; 3 Cancer Program, Broad Institute of MIT & Harvard, Cambridge, MA 02142, USA; 4 Mass General Brigham Department of Anesthesiology, Massachusetts General Hospital, Boston, MA 02129, USA; 5 Pulmonary and Critical Care Medicine, Brigham and Women’s Hospital, Boston, MA 02115, USA

**Keywords:** B cells, metabolic reprogramming, TME

## Abstract

B cells, an important component of adaptive immunity, play a key role in defense against a variety of infections and diseases including cancer. B cells play a dual role in cancer, contributing to both anti-tumor immunity and tumor progression. Metabolic reprogramming in the TME has a profound impact on B cell dynamics, reshaping their energetic landscape, influencing their differentiation and effector cell functions. These alterations arise from the complex interplay between intrinsic metabolic pathways and extrinsic factors, such as nutrient availability, hypoxic conditions, and tumor-derived signals. In the TME, B cells promote glycolysis over oxidative phosphorylation (OXPHOS) to meet the high energy demands of activation and proliferation. However, this metabolic plasticity is also mimicked by tumors, leading to dysfunctional B cell phenotypes, such as regulatory B cells (Bregs), which suppress anti-tumor immunity. Nutrient deprivation and accumulation of metabolic byproducts, including lactate, further impair B cell-mediated immune responses. This review highlights the complex interaction between B cell metabolism and cancer, emphasizing the effects of altered energetics on B cell function and implications on tumor progression and therapy. Decoding the metabolic vulnerabilities of B cells in the tumor niche can uncover novel therapeutic strategies against cancer.

## Simple Summary

B cells, essential components of adaptive immunity, exhibit dual roles in cancer by promoting both anti-tumor and tumor-supportive functions. Within the tumor microenvironment (TME), metabolic reprogramming reshapes B cell energetics, favoring glycolysis over oxidative phosphorylation to sustain activation and proliferation. However, tumor-induced metabolic alterations, such as nutrient deprivation and lactate accumulation, impair B cell function and promote immunosuppressive regulatory B cell phenotypes. This review examines how intrinsic and extrinsic metabolic factors influence B cell fate and function in the TME, highlighting metabolic vulnerabilities that can be targeted to enhance cancer immunotherapy.

## 1. Introduction

The maintenance of cellular homeostasis requires a coordinated balance between metabolic adaptability and immune competence. Immunometabolism has emerged as a critical field that investigates how metabolic processes shape immune cell function in pathological or physiological contexts. These processes are closely interlinked, highlighting the cross-talk between metabolic pathways and immune cells, including B cells that play an important role in anti-tumor defense. B cells are a critical component of the adaptive immune system, primarily responsible for humoral immunity. They play essential roles in immune defense by producing antibodies, presenting antigens to T cells, and secreting cytokines that regulate immune responses. Upon activation, B cells undergo clonal expansion and differentiate into antibody-secreting plasma cells or memory B cells, which provide long-term immunity [1,2]. Beyond their traditional roles in pathogen defense, B cells also contribute to immune regulation and tissue homeostasis. However, in the context of cancer, B cells exhibit a dual nature, acting as both anti-tumor effectors and facilitators of tumor progression. This functional plasticity is influenced by the TME, which imposes metabolic and functional constraints on B cells, altering their behavior and immune responses [3,4,5].

Metabolic alteration acts as a fundamental driver of immune cell function, dictating the ability of cells to meet the energetic and biosynthetic demands of activation, proliferation, and effector functions. Immune cells, including B cells, rely on dynamic metabolic reprogramming to adapt to environmental cues and functional requirements. For instance, during activation, B cells shift from a quiescent state, characterized by low metabolic activity, to a highly active state that demands increased glucose uptake, glycolysis, and oxidative phosphorylation (OXPHOS) [6,7,8]. This metabolic flexibility is essential for supporting the energy-intensive processes of antibody production, antigen presentation, and cytokine secretion [9]. However, in the TME, B cells are exposed to a metabolically hostile environment characterized by hypoxia, nutrient deprivation, and the accumulation of metabolic byproducts such as lactate [10,11]. These conditions disrupt normal metabolic pathways, leading to functional impairments and, in some cases, emergence of immunosuppressive phenotypes such as regulatory B cells (Bregs) [12,13,14].

Metabolic reprogramming is a hallmark of cancer, enabling tumor cells to sustain rapid proliferation, evade immune surveillance, and adapt to the harsh conditions of the TME. The most well-known example of this phenomenon is the Warburg effect, wherein cancer cells preferentially utilize aerobic glycolysis over OXPHOS, even in the presence of sufficient oxygen [15]. This metabolic shift not only supports the biosynthetic needs of tumor cells but also creates a nutrient-depleted immunosuppressive environment that affects the metabolism and function of immune cells, including B cells [16]. While much attention has been given to the metabolic reprogramming of T cells and macrophages in cancer, the metabolic adaptations of B cells remain underexplored [17,18]. Emerging evidence suggests that metabolic reprogramming in B cells is a critical determinant of their function in cancer, influencing their ability to mount effective anti-tumor responses or adopt tumor-promoting roles [19].

This review aims to provide a comprehensive overview of the metabolic reprogramming of malignant B cells and non-malignant tumor-infiltrating B cells in cancer, with a focus on the mechanisms driving these changes and their functional consequences. We focused our literature search based on published research articles and reviews relevant to B cell reprogramming, cellular plasticity, and associated signaling networks for potential therapeutic strategies. This review discusses the key metabolic pathways involved in B cell activation and differentiation, the impact of the TME on B cell metabolism, and the role of altered energetics in shaping B cell function in cancer. This work has expanded its scope to include the metabolic changes in tumor-infiltrating B cells in comparison to malignant B cells in the TME. Additionally, we will explore therapeutic opportunities to target B cell metabolism for improved cancer immunotherapy. By synthesizing current knowledge and identifying gaps in the field, this review seeks to highlight the importance of understanding B cell metabolism in the context of cancer and its potential as a therapeutic target. To provide context for how the TME reshapes B cell behavior in cancer, it is first essential to understand the core metabolic programs that govern normal B cell activation and differentiation.

## 2. Metabolic Pathways in B Cells: A Functional Overview

Glycolysis and oxidative phosphorylation (OXPHOS) play critical and dynamic roles in B cell activation and differentiation. Immature or naïve B cells are present in the spleen in the quiescent stage, and in this condition, they rely more upon oxidative phosphorylation compared to glycolysis for ATP production. Upon stimulation by antigens, naive B cells undergo metabolic reprogramming to increase glucose uptake and glycolysis to meet increased energy and metabolite demands, which is driven by the PI3K-AKT-mTORC1 pathway [20,21]. To reach their full metabolic potential, B cells require stimulation not only through the B cell receptor (BCR) but also through additional costimulatory signals and cytokines, which together are essential for driving complete B cell activation [22]. It has been reported that deletion of lactate dehydrogenase A (LDHA) leads to the induction of defective germinal center (GC) reaction [23]. In the initial stage of B cell activation in the germinal center, glucose-derived carbon may be preferentially diverted towards biosynthetic pathways such as the pentose phosphate pathway (PPP), while glutamine-derived carbon can replenish the TCA cycle and support mitochondrial respiration/OXPHOS [22,24,25]. Contrastingly, plasma blasts and plasma cells display elevated levels of both glycolysis and OXPHOS to meet the exceptionally high energetic and anabolic demands resulting from continuous antibody secretion [26,27].

In addition to the role of glycolysis and OXPHOS in B cell reprogramming, lipid metabolism emerges as an indispensable metabolic axis that supports B cell growth, signaling and effector function. B cells rely on lipids for membrane synthesis, and lipid rafts contribute to signaling and antibody production during activation. Different lipid metabolic pathways, such as de novo lipid synthesis, β-oxidation, and cholesterol metabolism, play an important role in B cell biology [28]. Upon activation, B cells take up fatty acids for clonal expansion and antibody production. CD36 and fatty acid binding protein (FABP) mediate lipid uptake in B cells to fulfill their energy demand [29,30]. mTORC1, SREBP, and a few other transcription factors involved in upregulating fatty acid synthesis in those cells by increasing expression of fatty acid synthase (FASN), acetyl-CoA carboxylase, after encountering an antigen and co-stimulatory signal [20,28,29]. De novo lipogenesis is more important than the presence of extracellular fatty acids for B cell proliferation and plasma cell differentiation [20,31,32]. Having high metabolic demand and hypoxic environment, germinal center B cells rely more upon fatty acid oxidation (FAO) [33]. For the survival and low-level antibody secretion of long-lived plasma and memory B cells supported by FAO [33,34]. B cell receptor and co-receptor clusters in the lipid rafts in the plasma membrane, which is organized by cholesterol and sphingolipids, which supports B cells signaling and fate decisions. After stimulation by LPS, TLR ligands, CD40, or BCR, B cells enhance cholesterol metabolism and LDL uptake for their proliferation and survival [35].

Further, amino acids are the building blocks of immunoglobulin, and, therefore, amino acid metabolism plays an important role in antibody production by B cells and plasma cells. Amino acid transport and synthesis in B cells are tightly regulated to support activation, proliferation, and antibody production [36,37]. Large neutral amino acids such as leucine, isoleucine, valine, phenylalanine, tryptophan, methionine, threonine, and histidine are transported through the System L transporter LAT1 (SLC7A5) with its partner CD98 (SLC3A2), which is elevated upon stimulation by LPS [35]. Uptake of leucine through LAT1-CD98 promotes mTORC1 signaling, which facilitates B cell growth, cell cycle entry, and differentiation towards antibody-secreting cells [35,36,38]. SLC1A5, SNAT1 (SLC38A1), and SNAT2 (SLC38A2) are important for transporting glutamine [36,39]. A lot of non-essential amino acids are synthesized from glucose and glutamine, which supports both antibody polypeptide synthesis and the energy demand of high-rate secretion [7,40,41]. BCR/TLR9-stimulated B cells activate mTORC1 signaling and effector response through production of branched chain amino acids by branched chain amnio acid aminotransferase (BCAT1) [42]. Asparagine is a key regulator of germinal center B cell homeostasis, and its deprivation leads to impaired germinal center reaction, affinity maturation, and humoral immunity [43]. Scarcity of amino acids reduces mitochondrial respiration and antibody secretion of plasma cells [7,38,44]. Bacteria-produced d-amino acids and mammalian d-amino acid catabolism together tune intestinal B cell survival and IgA class switching, thereby controlling IgA levels and maintaining balanced gut microbiota [45].

**Figure 1:**
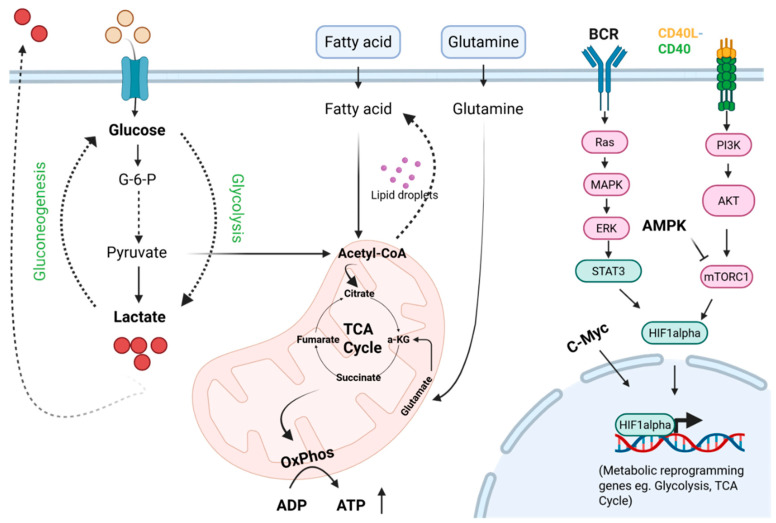
Schematic overview of the major metabolic pathways operating in B cells. The image depicts the major metabolic pathways in B cells such as glycolysis, the tricarboxylic acid (TCA) cycle and mitochondrial oxidative phosphorylation (OXPHOS), fatty acid metabolism, and amino acid (e.g., glutamine) metabolism. Key signaling regulators such as mTORC1, AMPK, c-Myc, and HIF-1α are depicted that link extracellular cues to metabolic reprogramming. [Created with BioRender.com].

Plasma cells and memory B cells use similar core metabolic pathways including glycolysis and oxidative phosphorylation, though they use them at different levels to match their distinct functional requirements. Plasma cells require high energy for the continuous secretion of antibodies, whereas memory B cells have low metabolic demand and flexible metabolic activity to support their long-term survival and quick memory response [26,46,47]. In the bone marrow, plasma B cells have a high uptake capacity of glucose, amino acids, fatty acids and vitamins for their rapid immunoglobulin secretion and ER expansion [26,46,48]. Those plasma cells have elevated expression of glycolytic and TCA cycle enzymes that lead to high glycolytic and oxidative phosphorylation rates which support their protein synthesis and ER maintenance. Transcriptional regulation of XBP1 increases phosphatidyl choline synthesis, which is utilized in ER and membrane lipid synthesis. In addition, glucose is also utilized for the hexosamine pathway for glycosylation of antibody. BLIMP-1 regulates the expression of amino acid transporters and increased uptake of amino acids (leucine, arginine) activate mTORC1 signaling which promotes antibody secretion [20,26,49,50,51]. Memory B cells maintain low glycolytic and oxidative phosphorylation activity to meet basal cellular needs and preserve redox balance, however their metabolic demand rises rapidly upon reactivation after antigen encounter, enabling differentiation into antibody secreting cells [24,46,47]. A schematic representation of the various metabolic pathways that occur in B cells is given in Figure 1. These observations highlight that B cell subsets adopt distinct metabolic programs to support their specialized functions, from antibody production in plasma cells to rapid recall responses in memory B cells. However, these intrinsic metabolic pathways do not operate in isolation. In cancer, they are profoundly influenced by the TME, where nutrient competition, hypoxia, stromal interactions, and soluble tumor-derived factors can reprogram B cell metabolism and ultimately alter their differentiation, survival, and immune function.

## 3. TME and Its Role in Altered B Cell Metabolism

The TME comprises of a complex and diverse ecosystem made up of tumor cells, which are themselves heterogenous, along with cancer associated fibroblasts, macrophages, endothelial cells, and different immune cells. Since tumor cells proliferate rapidly and consume nutrients at a high rate, the microenvironment is deprived of key metabolites such as glucose, pyruvate, free fatty acids, glutamine and other amino acids. Additionally, poor vascularization and tumor-driven glycolysis, make the TMEs hypoxic in nature and lead to extracellular lactate accumulation.

In tumors, the majority of the infiltrating cells are T lymphocytes, while B cells represent a minor fraction; however, this observation could be contextually different in hematological malignancies of B cells [52,53]. The tumor cell or the microenvironment cells secrete several chemoattractants that induce B cell migration into the TME [54,55,56]. After the B cells enter the tumor site, they can interact with neo antigens that can lead to their migration to the secondary lymphoid structures or tertiary lymphoid organs. B cell activation and proliferation happen mostly in the germinal centers of the secondary lymphoid organs. However, similar temporary tertiary lymphoid organs (defined as the TLS) formed close to the tumor site where the tumor draining lymph can accumulate. Although the presence of the TLS is associated with better patient prognosis, many tumors with B cell infiltrate display disorganized or no TLS, suggesting an alternative expansion or activation of B cells influenced by the TME. Table 1 depicts the classification of B cell subtypes and their role in tumors and metabolic regulation.

In vitro studies show that breast cancer cells produce leukotriene B4 (LTB4), a lipid metabolite generated through the 5-lipoxygenase signaling pathway. LTB4 engages peroxisome proliferator-activated receptor-α (PPARα) expressed on B cells, driving the differentiation of regulatory B cells (Bregs) that contribute to tumor immune evasion [68]. L-glutamine has been known for a while to be essential for both proliferation, plasma cell differentiation, and Ab production of human B cells [69]. In studies, supported by in vitro human cell line and in vivo murine models, it was shown that cancer cells show high glutamine consumption, [70], thus limiting the availability of glutamine for the differentiating B cells in the TME and in the TLS, thereby dampening antitumor immune responses. On the contrary, Ma et al. demonstrated that there is an alternative pathway for B cell differentiation, that is glutamine dependent, leading to atypical memory B cell formation (AtMs). These AtMs have lower BCR maturation, recognize healthy tissue, are functionally defective, and expressed markers such as CD11c, FCRL4 (Fc receptor-like protein 4), and T-BET [63]. Interestingly, glutamine dependency changes with cancer stages [71,72,73] and the availability of glutamine may impact AtM production or inhibition of B cell differentiation during different cancer stages. Recently Aryl hydrocarbon receptor (AhR) signaling has been associated with IL-10-producing CD21^hi^CD24^hi^ Breg differentiation [74]. Cancer cells can produce AhR ligand (like Kynurenine) by hijacking the tryptophan metabolism pathway enzymes such as IDO, TDO, and AFMID [75,76,77]. The AhR-dependent orientation of B cells into IL-10 Breg is correlated with the inhibition of GC and plasmablast differentiation and subsequently with reduction of the pro-arthritogenic response [78]. Similarly, Trp metabolizing enzymes like IDO-1 activity in B cells are associated with immunosuppression through Breg induction [79,80]. Short chain fatty acids (SCFA), like butyrates have been associated with AhR induction and Breg production. However, cancer cells are mainly consumers of SCFAs, which are physiologically produced by certain microbial fauna [78,81].

**Figure 2:**
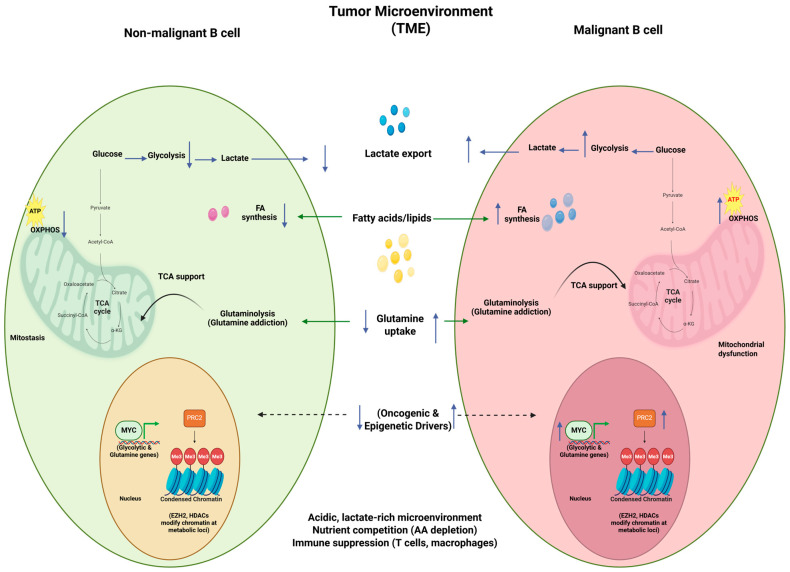
Metabolic reprogramming of non-malignant B cells and malignant B cells in the TME. In comparison to non-malignant B cells, malignant B cells increase glucose uptake and glycolysis, exporting lactate that acidifies the TME and suppresses immune responses. They show reduced reliance on oxidative phosphorylation, enhanced fatty acid uptake and synthesis, and addiction to amino acids such as glutamine and branched-chain amino acids, which activates mTORC1 signaling and depletes nutrients in the microenvironment. These metabolic shifts are driven by oncogenic and epigenetic regulators (e.g., MYC, PI3K, EZH2, HDACs) and collectively support tumor growth, survival, and immune evasion. [Created with BioRender.com].

Tumor cells induce regulatory B cells (Bregs) through both cytokine secretion and direct cell–cell interactions. The PD-L1/PD-1 signaling pathway has emerged as a critical regulator of Breg differentiation and suppressive function. Notably, PD-L1–expressing human breast cancer epithelial cells promote the generation of CD24^+^CD38^+^ Bregs upon co-culture with B cells, a population linked to advanced tumor grade and increased Treg prevalence in invasive breast cancer patients [82]. Studies in both murine models and human pancreatic cancer samples reveal that tumor-derived IL-18 induces the development of PD-1–expressing regulatory B cells. These Bregs exert immunosuppressive effects by inhibiting cytotoxic T cells and NK cells through IL-10–mediated mechanisms [83]. Tumor-derived extracellular vesicles play a key role in Breg induction. Exosomes from oesophageal squamous cell carcinoma patients contain TLR4- and MAPK-associated mRNAs that drive the generation of IL-10^+^ and PD-1^+^ Bregs from healthy CD19^+^ B cells [84]. Similarly, hepatocarcinoma-derived exosomes enriched in HMGB1 promote the expansion of IL-10–producing TIM-1^+^ Bregs, a population associated with advanced tumor stage and poor patient survival [85]. The dynamics of metabolic reprogramming in B cells in the TME is depicted in Figure 2. Together, these findings show that tumor-derived signals, including extracellular vesicles, not only skew infiltrating B cells toward immunosuppressive Breg phenotypes but also reshape their metabolic state within the TME. This broader interplay between tumor cues, immune regulation, and metabolic adaptation provides the context for understanding metabolic reprogramming in cancer, where malignant B cells adopt distinct bioenergetic programs, most notably enhanced aerobic glycolysis, to support growth, survival, and immune evasion.

## 4. Metabolic Reprogramming of B Cells in Cancer

The most prominent metabolic reprogramming in B cell malignancy is its preference towards glycolysis whereby it produces lactate in aerobic conditions which mimics the Warburg effect. Increased glycolytic activity provides ATP rapidly, thus maintaining redox balance by producing NADPH through pentose phosphate pathway and synthesize nucleotide and amino acid precursors to promote invasion and immune evasion [20,22]. Compared to the activated B cells, malignant B cells have chronic-oncogenic signaling (MYC, PI3K) which drives them into hyper-glycolytic state. Malignant B cells have high expression of glucose transporters (GLUT1/3) and glycolytic enzymes (HK2, PFKB3, LDHA). LDHA involved in lactate production from pyruvate and elevated LDHA promotes cell survival through STAT5 and ERK signaling in diffuse large B cell lymphoma (DLBCL) [86]. The transcription factors such as PAX5 and IKZ51 suppress glycolytic branching pathway in normal B cells; however, in malignant B cells, there is an upregulation of different glycolytic branching pathways [87,88]. Elevation of glucose-6-phosphate dehydrogenase (G6PD) in pentose phosphate pathway (PPP) synthesizes NADPH to maintain redox balance and also produces ribose-5-phosphate, which is a precursor for nucleotide synthesis. The hexosamine biosynthetic pathway (HBP) converts fructose-6-phosphate into UDP-GlcNAc for glycosylation of cell surface and oncogenic signaling proteins. One of the rate- limiting enzyme GFPT1, in HBP is upregulated and leads to an increase in glycosylation of PD-L1 and, therefore, support tumor growth via immune escape, which is relevant in B cell malignancy [89,90]. Another glycolytic product, 3-phosphoglycerate (3-PG) is involved in the serine /glycine pathway to supply the high serine demand of malignant B cells for the production of one-carbon metabolites and glutathione, to tolerate oxidative stress in B cell tumors [91,92].

Malignant B cells have glycolytic activity and Warburg like phenotype, and they are less dependent upon oxidative phosphorylation and mitochondrial function. The protein expression of complex 1 and other respiratory complex is less, and their functional activity is impaired in B cell tumors [93,94]. In DLBCL, a subset of tumors are high glycolytic, which are BCR dependent, whereas OxPhos-DLBCLs are BCR -independent and they have much higher ETC activity and mitochondrial energy metabolism [95]. Metabolic reprogramming in B cells complexes pyruvate for fueling glutaminolysis, which in turn is involved in main complexes to sustain biosynthesis and aneplerosis [96,97]. B cell lymphoma has altered mitochondrial apoptotic pathway, which is driven by BCL2 family proteins such as BCL-2, MCL-1, or BCL-XL, which are anti-apoptotic to maintain mitochondrial outer membrane permeabilization and cytochrome c release [98,99].

Malignant B cells exhibit elevated fatty acid and cholesterol synthesis to support their rapid proliferation and survival. Fatty acid synthase (FASN) is markedly overexpressed, with its expression levels serving as an indicator of poor prognosis among B cell malignancies [100,101]. Compared to normal B cells, CLL has STAT3-dependent CD36 overexpression, which facilitates fatty acid uptake from the TME [102]. Targeting fatty acid metabolism is vulnerable to different subtypes of DLBCL cells [103]. Lack of tetraspanin CD37 in aggressive B cell lymphoma increases uptake, storage, and oxidation of exogenous fatty acid to sustain in lipid-rich niches like lymph nodes [104]. Like most of the cancer cells, B cell lymphoma cells are addicted to glutamine to enable their rapid proliferation in nutrient deprived environment. Depending upon their nutrient utilization capacity, glutamine addicted B cell lymphoma has higher oxygen consumption, and they are resistant to glycolytic inhibition [105]. Oncogenic driver MYC regulates the overexpression of glutamine transporter (SLC1A5, SLC7A5) and glutaminase (GLS). However, accumulation of α-ketoglutarate induces ROS generation and leads to ferroptosis in DLBCL [106]. In addition to glutamine metabolism, B cell lymphoma has elevated expression of enzymes (PHGDH, PSAT1, PSPH) involved in serine biosynthesis, followed by production of glycine, whereas overexpression of SHMT2 maintains the redox balance and nucleotide biosynthesis in DBLCL [107,108]. In this type of cancer, there is increased uptake of branched-chain amino acids (BCAA) (leucine, isoleucine, and valine) through LAT1/SLC7A5 and activation of mTORC1 for their proliferation, and eventually this leads to decreased availability of BCAA in the plasma of the TME [107,109]. Tryptophan metabolism generates two immunosuppressive product kynurenine (Kyn) and kynurenic acid, in DBLCL [110]. Elevated arginase (ARG1/2) produces ornithine and urea to support B-ALL proliferation, along with T cell suppression in the TME [111,112,113]. Arginine is also converted to citrulline and nitric oxide by nitric oxide synthase 2 (NOS2), which exerts an antitumor effect by enhancing M1 macrophage polarization and apoptosis in blasts [114].

Epigenetic modifications, such as histone modification, DNA methylation, and long- non-coding RNA, regulates the expression of genes involved in glycolysis, oxidative phosphorylation, or amino acid metabolism, and, therefore, they determine the fate of B cells [115]. One of the histone modifiers HDAC, deacetylases at glycolytic loci, repressing transcription during quiescent phases, whereas HAT acetylates H3K27ac at the promoter of GLUT1 and HK2 for the activation of B cells. To silence c-Myc-driven glycolysis in plasma cells, HDAC is recruited by Blimp1 [115,116]. EZH2/PRC2 complex represses metabolic inhibitors by methylation of H3K27me3 and activates glycolytic genes OXPHOS in plasma cells, while BCL6 represses catabolic programs in germinal center B cells. Abnormal EZH2 elevation in B cell lymphomas promotes high glycolysis via de-repression of PFKFB3 [117]. Hypermethylation of the ASNS promoter by DNMT1 silenced the activation and, therefore, increased the sensitivity to ASNase in B cell malignancy [118]. During GC reaction, CpG islands of nutrient transporters are demethylated by TET2/3. Whereas cMYC recruits TET for upregulation of arginine synthesis genes under stress conditions [115,119]. SIN3 represses glycolytic genes (PFK, ENO) by histone deacetylation [120].

During the rapid proliferation of naïve and germinal Centre B cells, cMYC promotes glycolysis and glutamine uptake by upregulating GLUT1, HK2, LDHA, GLS/SLC1A5 after stimulation by BCR/CD40. EBF1 and E2A activate nutrient transporters like SLC7A5 (LAT1) for essential amino acids, supporting mTORC1 and protein synthesis early in B cell development [20,121]. BCL6 and EBF1 repress oxidative metabolism for biosynthetic shifts, with dysregulation promoting lymphoma via arginine/polyamine pathways [20,115]. BCL6 also represses mitochondrial genes (PPARα), OXPHOS, and shifts to lipid synthesis for dark zone proliferation. Blimp-1 (PRDM1) silences c-MYC and glycolytic enzymes during plasma cell differentiation, shifting to secretory metabolism with high Ig glycosylation demands [20,38]. In B cell cancers, hyperactive c-MYC upregulates BCAT1 (BCAA), ASNS (asparagine), and ASS1 (arginine) pathways, amplified by aberrant Notch/HIF1α [119,122,123]. Collectively, these transcriptional regulators coordinate the metabolic programs that determine whether B cells support rapid proliferation, germinal center cycling, or plasma cell differentiation, while their dysregulation in malignant settings drives nutrient dependence and metabolic plasticity. Thus, understanding how altered metabolic circuitry translates into changes in B cell behavior provides the basis for examining the functional consequences of altered energetics in B cells.

## 5. Functional Consequences of Altered Energetics in B Cells

Altered cellular energetics reshape virtually every stage of B cell biology. Naïve B cells are metabolically quiescent and, upon antigen encounter, must rapidly upregulate glycolysis, oxidative phosphorylation (OXPHOS), and nutrient uptake to sustain clonal expansion and germinal center (GC) formation. Disruption of key metabolic nodes (for example, mTORC1 signaling, GLUT1-dependent glucose import, LDHA-driven aerobic glycolysis, or mitochondrial remodeling) blunts B cell receptor (BCR)–induced growth and cell cycle entry, leading to defective GC reactions and skewed fate decisions toward short-lived plasmablasts or apoptosis instead of high-affinity memory and long-lived plasma cells [23,46,47]. Additionally, metabolic cues integrate with transcriptional and epigenetic programs that determine B cell differentiation. Altered availability of TCA intermediates such as α-ketoglutarate, acetyl-CoA, or S-adenosylmethionine modifies the activity of demethylases and acetyltransferases, thereby affecting expression of lineage-defining factors like BCL6 and BLIMP1 and ultimately diverting cells away from optimal GC or plasma-cell fates [38,47].

Plasma cells require high mitochondrial respiration, amino acid uptake, and ER-supporting biosynthesis to sustain massive antibody secretion; energetic insufficiency limits immunoglobulin synthesis, promotes ER stress, and predisposes to plasmablast death [47]. Conversely, nutrient excess or chronic activation of anabolic pathways can drive low-affinity, short-lived plasma cell responses at the expense of durable humoral memory, contributing to qualitatively poor antibody responses in chronic infection, obesity, or aging [124]. Antigen presentation by B cells depends on ATP-demanding processes including antigen uptake, endosomal processing, MHC class II loading, and expression of co-stimulatory molecules. When glycolysis or OXPHOS is constrained, B cells display reduced MHC II [125] and costimulatory molecule expression CD80/CD86, diminished cytokine production, and impaired ability to prime or sustain CD4 T cell responses, weakening T-dependent antibody responses and favoring immune evasion [13,38,46].

Regulatory B cells (Bregs) rely on distinct metabolic wiring compared with conventional effector B cells, often favoring oxidative metabolism and lipid utilization to support IL-10 production. Conditions that limit glucose availability or chronically activate stress-responsive pathways can preferentially support Breg differentiation or stabilization, enhancing their capacity to secrete IL-10, TGF-β, and other mediators that suppress effector T cells, dendritic cell activation, and inflammatory cytokine cascades. In cancer and chronic inflammation, tumor-derived metabolites (lactate, kynurenine, adenosine) and hypoxia further skew B cell metabolism toward a Breg-like phenotype. This metabolically driven expansion of Bregs contributes to local and systemic immunosuppression, dampens anti-tumor T cell responses, and may undermine vaccine efficacy by suppressing productive GC reactions [38,58,124].

Altered B cell energetics disrupts productive interactions with T follicular helper (Tfh) cells by reducing expression of co-stimulatory molecules, cytokines, and chemokine receptors required for efficient GC synapse formation and selection. Metabolic stress can also impair expression of CXCR5, CCR7, and other guidance cues, disorganizing B cell positioning within follicles and GCs, thereby perturbing the spatial coordination needed for effective affinity maturation [126]. Reciprocally, metabolic rewiring of B cells feeds back onto dendritic cells and T cells through altered cytokine and antibody profiles. Energy-stressed or Breg-skewed B cells tend to produce more IL-10 and less pro-inflammatory cytokines, inhibit DC maturation, constrain CD4 and CD8 effector responses, and favor T cell exhaustion or Treg expansion, reshaping the overall immune network toward tolerance rather than robust immunity [127]. Recognizing this broader immunoregulatory impact provides a strong rationale for therapeutically targeting altered B cell metabolism, with the aim of restoring effective antitumor immune responses and overcoming immune suppression within the TME.

## 6. Therapeutic Opportunities in Altered B Cell Metabolism

We discuss different therapeutic strategies targeting malignant and non-malignant tumor-infiltrating B cells in TME.

### 6.1. Therapeutic Approaches for Targeting Metabolic Alteration in Malignant B Cells

Metabolic targeting of malignant B cells (B Cell lymphoma and multiple myeloma) is in clinical use, although most of the drug’s target signaling or stress pathways lie upstream of metabolism rather than single metabolic enzymes.

(A) Targeting mTORC1/mTORC2 pathway

It has been discussed that mTORC1 is hyperactivated in DLBCL, follicular lymphoma, and mantle cell lymphoma, and that it regulates anabolic growth, glycolysis, and lipid synthesis [96,128].

A different type of mTORC1 inhibitor (Rapalog) has been used to reduce protein/lipid synthesis and glycolysis in lymphoma cells, such as temsirolimus, everolimus, and sirolimus. Temsirolimus is approved for relapsed mantle cell lymphoma (MCL), whereas it is also evaluated in relapsed DLBCL and other non-Hodgkin lymphoma (NHL) in phase 2 studies. Similarly, everolimus has been tested in phase 1/2 trials for relapsed DLBC and MCL. This drug could be used as a single agent or in combination [129]. Vistusertib (AZD2014) is a second-generation mTORC1/2 inhibitor that is in early-phase hematologic trials. In DLBCL, it synergistically suppresses tumor growth in the xenograft model in combination with PI3Kβ/δ inhibitor AZD8186 [130,131].

(B) Targeting PI3K-AKT-mTOR pathway

PI3K is a key upstream regulator of glucose uptake, glycolysis, and survival in malignant B cells. Class I PI3K inhibitors (idelalisib, copanlisib, duvelisib, and umbralisib) are approved or tested in indolent lymphomas and CLL. Idelalisib is a PI3Kδ inhibitor approved for relapsed follicular lymphoma, small lymphocytic lymphoma, and CLL [132]. Copanlisib is a pan-class I PI3K inhibitor with α/δ bias, which is approved for relapsed follicular lymphoma. Multiple NHL trials are completed or ongoing with this drug [133]. Like copanlisib, duvelisib, and umbraisib, also used in clinical trials for NHL and CLL. A potential drug candidate targeting PKCβ signaling, used in clinical studies is Enzastaurin (LY317615.HCl) and has been reported to induce apoptosis and inhibit proliferation in multiple B cell malignancies [134,135].

(C) Targeting amino acid and lipid metabolism

Selective inhibition of glutaminase (GLS), such as telaglenastat, displays preclinical efficiency in glutamine addicted multiple myeloma and lymphoma and are currently under consideration as a therapeutic agent in combination with checkpoint inhibitors and chemotherapy [136,137,138]. Arginine-depleting enzymes, including L-asparaginase and drugs that target SLC7A11, SLC7A5, or SLC1A3 are currently in consideration for utilizing amino acid dependency in leukemia and lymphoma affecting various immune effector cells including B cells [71,137,139].

Low dose statins that inhibit HMGCR combined with rapamycin, which inhibits mTOR signaling, have synergistic effects blocking SREBP signaling, lowers lipid rafts and dampens B cell lymphoma proliferation, highlighting the SREBP-mTOR axis as an effective target for therapy in DLBCL models [140].

(D) Targeting mitochondrial dynamics and inducing apoptosis

Emerging therapeutic strategies in B cell cancer directly target mitochondria to kill cancerous B cells through OXPHOS. Small molecules like BTM 3528 and BTM 3566 have been reported to hyperactivate mitochondrial stress response by activating inner membrane protease, OMA1, thereby resulting in DELE and OPA1 cleavage, mitochondrial fragmentation and ultimately apoptosis in diffuse large B cell lymphoma (DLBCL) model systems with complete regression seen in patient-derived xenografts [141]. Further, mitochondrial-specific Sirtuin 3 (SIRT) inhibitors like SJ 106C and its predecessor, YC8 02 accumulate in the mitochondria and inhibit SIRT3 deacetylase activity, thus perturbing mitochondrial metabolism, elevating oxidative stress and reducing DLBCL growth [142]. Mitochondria targeted pro-apoptotic strategies, for instance, enhancing mitophagy, induction of mitochondrial permeability and ROS burden are promising and being considered for early-phase clinical trials. Although not all these strategies are B cell specific, DLBCL and B cell lymphomas are consistently highlighted as leading candidates where mitochondria and OXPHOS directed therapies would be an efficient approach.

### 6.2. Therapeutic Strategy for Targeting Tumor-Infiltrating B Cells (TIL-B Cells) in Cancer

Tumor-infiltrating B cells are heterogeneous in populations, with mainly two groups: pro-immune B cells, which are antigen-presenting cells to CD4 and CD8 T cells, and they secrete pro-inflammatory cytokines that support T cell responses. Regulatory B cells secrete IL-10, TGF-β, and other mediators that support angiogenesis and tumor growth [143,144,145]. This dual behavior makes it risky to target beneficial B cells along with suppressive ones.

There are currently no clinical trials that specifically target TIL B cells, but existing human studies are indirect or agonistic to B cell subsets. However, there is a clinical trial ongoing that targets tumor associated lymphocytes. Current clinical trials fall into broader immunometabolism or TME -modifying categories that unavoidably affect TIL-B metabolism, but without B cell focused endpoint.

Global tumor metabolism modulators:

BPM31510, CoQ-modulating formulation targets oxidative phosphorylation or mitochondrial function, is in early-phase trials across solid tumors and changes the metabolic landscape for all infiltrating immune cells, including B cells [146]. There are a few trials ongoing targeting amino acid metabolism (e.g., glutaminase inhibitor NCT03872427 or arginase inhibitor, INCB001158) alters the nutrients for T cells, myeloid cells, and B cells, but do not stratify outcomes by TIL-B phenotype [147,148,149,150].

B cell–directed trials in cancer (e.g., anti-CD20, BTK inhibitors, CD40 agonists) are generally focused on B cell number and function rather than their metabolic wiring, and are not framed as “metabolism-targeting” even when they alter metabolic signaling pathways [151,152,153,154].

Current drugs under consideration and combination strategies that target B cells have been listed in Table 2.

## 7. Challenges and Future Directions

Studies on how metabolic reprogramming dictates B cell behavior in cancer states are still ongoing. These investigations are facing challenges primarily because tumor-infiltrating B cells are highly heterogenous and can switch states over time, metabolism is highly influenced by nutrient conditions that are highly variable across tumor regions and disease stages, and several of the metabolic pathways are shared between immune cells and cancer cells, thus making it difficult to target metabolic pathways without collateral effects on normal cells. Emerging technologies such as single-cell, spatial, and perturbation approaches are being extensively explored to enable more precise, actionable models of B cell metabolic states in tumors. As discussed earlier, the TME contains diverse B cell subsets and bulk assays often miss the subset-specific programs in B cells. Single-cell RNA sequencing (scRNA-seq) can overcome this diversity problem by profiling thousands of individual B cells from tumors. While this technique cannot quantify metabolites directly, it can efficiently specify the expression levels of metabolic enzymes, transporters and signaling hubs, for example, glycolysis, amino acid transport, lipid metabolism pathways and mTOR/AMPK signaling. Computational pathway scoring can then be used to nominate distinct metabolic modes that are linked to metabolic activity, antibody secretion, or immunosuppressive B cell phenotypes. Further, integration of scRNA-seq with BCR sequencing can connect metabolic programs to clonal expansion, antigen experience, and differentiation trajectories. Alternatively, single-cell metabolomics can be used to directly quantify metabolites and quantify flux using mass spectrometry-based approaches with metabolic probes. This may be followed by paired multi-omic profiling approaches in matched patient samples to elucidate transcription-inferred metabolism, post-transcriptional regulation, and correlate B cell metabolic states to clinical outcomes and patient response to immunotherapy.

Since, B cell metabolism is highly context dependent, proximity to blood vessels, stromal niches, tertiary lymphoid structures, or hypoxic tumor cores determines nutrient access and accumulation of waste. Spatially resolved tools can preserve tissue architecture and reveal how location shapes B cell fate. Intravital and multiphoton microscopic techniques combined with the use of fluorescently labelled metabolic reporters, for instance, glucose/lactate sensors or NADH/FAD-based redox readouts can track B cell movement and metabolic shifts in real time in both preclinical murine and in vitro models. Spatial transcriptomics and imaging mass cytometry techniques can map metabolic enzymes and signaling pathways in the tumor niche while retaining phenotypic identity. In the clinical setup, PET and metabolic tracers, including glucose uptake tracers and emerging probes for amino acid and lipid sensing, can help in the identification of metabolically active immune niches. Current efforts are focused on improving resolution, multimodal workflows integrating metabolic phenotypic and functional relationships, and B cell selective probes to selectively study B cell metabolism within mixed tissues.

However, metabolic profiling alone cannot determine which metabolic pathways determine B cell states in cancer. CRISPR-based approaches enable systematic study of metabolic dependencies, targeting pathways, including glycolysis, mitochondrial function, amino acid transport, lipid metabolism, and signaling pathways, like mTOR, AMPK, MYC, and HIF-1. The strongest future strategy involves a combinatorial approach, that integrates CRISPR interventions with single-cell and spatial technologies that could potentially correlate specific genetic alterations to metabolic rewiring, lineage decisions, and tissue localization.

Thus, single-cell multi-omics spatial metabolic imaging and CRISPR-based perturbation platforms are rapidly being explored to define B cell metabolic programs in cancer and identify causal targets for therapeutic intervention. Translating these insights into effective therapeutic approaches will require a separation of tumor versus immune metabolic requirements, strategies to limit systemic toxicity, and accounting for patient-to-patient variability in the TME. Longitudinal patient cohorts and reliable preclinical models- analyzed with integrated multimodal pipelines will be essential for investigating metabolic interventions that could potentially enhance anti-tumor B cell functions while inhibiting pro-tumor regulatory programs.

## 8. Conclusions

B cells are now gaining attention as versatile regulators of cancer immunity, capable of both, inhibiting and promoting tumor growth. B cell function is tightly linked to their metabolic state. In normal circumstances, B cells shift from a quiescent, energy efficient state to a highly active one supporting proliferation, antibody synthesis, and memory. This homeostasis is dysregulated in cancer. TME is hypoxic, nutrient-depleted and rich in lactate and kynurenine. This disrupts and limits antigen presentation and antibody responses, while favoring regulatory B cells that reduce anti-tumor immunity.

Intrinsic oncogenic signals further reprogram metabolism to favor increased glycolysis, altered mitochondrial function and enhanced dependence on amino acids and lipids in the context of B cell malignancies. Understanding the mechanisms underlying this metabolic reprogramming sheds light on strategies and targets for therapeutic intervention. Concurrently, non-malignant B cells inside the TME can either favor anti-tumor T cell responses or lead to immune suppression, depending on their metabolic dynamics.

Therapeutic strategies modulating B cell metabolism exploit these insights for effective cancer immunotherapy. Approaches that target glycolysis, mitochondrial dynamics, lipid and amino acid metabolic pathways are being extensively explored, often in combination with established treatment approaches such as kinase inhibitors, monoclonal antibodies, and immune checkpoint blockade. The primary challenge is to selectively limit pathogenic or malignant B cell populations while preserving beneficial, tumor-reactive B cells. Ongoing studies are focusing on better defining B cell metabolic states in human cancers and integrating metabolic interventions with rational combination strategies. Taken together, investigating and modulating B cell energetics offers a promising approach to precise and durable cancer immunotherapy.

## Figures and Tables

**Table 1:** B cell classification and their function in the TME.

B Cell Type	Main Function in Tumors	Metabolic/Functional Regulation
GC-like B cells (GCBCs) in TLS	Undergo affinity maturation and class switching to generate high-affinity tumor-specific antibodies; support local T follicular helper (Tfh) and CD8^+^ T cell responses [52,56,57]	Sustained activation and proliferation require high metabolic activity supported by cMyc, BCL6, and PKM2. Glucose maintains PPP pathway and one carbon metabolism. While FFA, glutamine and asparagine fuel the TCA cycle for ATP production [25,47,58,59]. GCBCs are supported by CXCL13^+^ stromal/Tfh niches and antigen availability [56,60].
Memory/activated B cells	Present tumor antigens to CD4^+^ and CD8^+^ T cells, produce proinflammatory cytokines (e.g., TNF, IFNγ), and help to recruit other immune cells via chemokines (CCL3, CCL4, CCL5, CXCL10, CXCL13) [55,56].	Metabolically quiescent. Low Akt-mTOR signaling and FOXO1/PAX5 signaling maintains self-renewal and low metabolic state [58,61]. Activation signals (BCR, CD40, TLR) and inflammatory cytokines enhance metabolic demand, promoting glycolysis and costimulatory molecule expression (CD80/CD86, CD40) that sustain T cell activity [55,56,62].
Plasma cells (antibody-secreting)	Produce IgG/IgA against tumor antigens, mediating ADCC, ADCP, and complement activation; often associated with favorable prognosis and response to immunotherapy.	High-rate antibody secretion depends on strong anabolic metabolism and nutrient supply from the TME; chronic antigen stimulation and survival factors (BAFF, APRIL) promote their maintenance in TLSs or tumor stroma. BLIMP1, mTORC1, and c-Myc drive anabolic growth and high OXPHOS [27,58].
Atypical memory B cell (AtMs)	They are mostly low reactive towards tumor cells and produce autoreactive antibodies.	Glutamine is important for their differentiation [63].
Regulatory B cells (Bregs)	Suppress antitumor immunity via IL-10, IL-35, TGFβ, PD-L1, CD39/CD73; promote Treg expansion and M2 macrophage polarization, facilitating tumor progression [56,64,65].	Tumor-derived cytokines, chronic antigen exposure, drive conversion of conventional B cells into Bregs and support their survival in immunosuppressive niches [64]. Besides metabolic stress (e.g., hypoxia, metabolites) can also induce Bregs [66,67].

**Table 2:** Therapeutic Landscape of combination therapy targeting B cells.

B Cell Target	Drug Name	Combination Strategies in Clinical Trials or in Practice
PD-1 on B cells (and T cells)	Pembrolizumab, nivolumab, cemiplimab	Frequently combined with anti-CD20 mAbs (rituximab, obinutuzumab) or BTK inhibitors (ibrutinib, acalabrutinib) in B cell lymphomas to enhance T cell and B cell effector functions [155].
PD-L1 on Bregs and other B cells	Atezolizumab, durvalumab, avelumab	Studied in combination with anti-CD20 antibodies and chemotherapies (e.g., R-CHOP-like backbones) in aggressive B cell lymphomas to overcome PD-L1–mediated immune suppression [156,157].
CTLA-4 axis (via CD80/CD86 on B cell APCs)	Ipilimumab, tremelimumab	Evaluated mainly in solid tumors to involve T cells but conceptually combined with PD-1/PD-L1 inhibitors and anti-CD20 therapies to augment co-stimulation from B cell antigen-presenting cells in the TME [158,159,160].
IL-10-producing Bregs	No Breg-specific drug yet; indirect targeting via ibrutinib, lenalidomide, and PI3Kδ inhibitors (idelalisib, umbralisib)	BTK and PI3Kδ inhibitors in combination with anti-CD20 antibodies (e.g., ibrutinib + rituximab) can reduce Breg-like signaling; lenalidomide + rituximab combinations modulate cytokine milieu and B cell subsets [161,162,163].
TGF-β-producing Bregs	TGF-β receptor/ligand inhibitors (e.g., fresolimumab, galunisertib—mostly solid tumor settings)	Combined with PD-1/PD-L1 blockade in early-phase trials in solid cancers; mechanistically relevant to B cell rich TMEs, although lymphoma-specific data remain limited [164,165].
Antibody- producing plasma cells (tumor-reactive)	Rituximab, obinutuzumab, ofatumumab, tafasitamab (CD19), polatuzumab vedotin (ADC), loncastuximab tesirine (ADC)	Multiple chemo-free or reduced-chemo regimens combine anti-CD20 or CD19 antibodies with lenalidomide or other targeted agents (e.g., tafasitamab + lenalidomide + R-CHOP in frontMIND) to harness and redirect humoral responses [166,167].
Metabolic checkpoints (adenosine, hypoxia, lactate) impacting B cells	SMI targeting adenosine pathway (e.g., CD73/A2A inhibitors in early trials), IDO1 inhibitors; not yet B cell-specific	Conceptual and early-phase strategies combine metabolic drugs (adenosine-axis or IDO1 inhibitors) with PD-1/PD-L1 blockade to reshape the TME, which includes metabolically suppressed B cells [168,169,170,171,172].

## Data Availability

No new data were created or analyzed in this study. Data sharing is not applicable to this article.
